# *LAM* Genes Contribute to Environmental Stress Tolerance but Sensibilize Yeast Cells to Azoles

**DOI:** 10.3389/fmicb.2020.00038

**Published:** 2020-01-28

**Authors:** Svyatoslav S. Sokolov, Margarita A. Vorobeva, Alexandra I. Smirnova, Ekaterina A. Smirnova, Nataliya I. Trushina, Kseniia V. Galkina, Fedor F. Severin, Dmitry A. Knorre

**Affiliations:** ^1^Department of Molecular Energetics of Microorganisms, Belozersky Institute of Physico-Chemical Biology, Lomonosov Moscow State University, Moscow, Russia; ^2^Faculty of Bioengineering and Bioinformatics, Lomonosov Moscow State University, Moscow, Russia; ^3^Department of Neurobiology, University of Osnabrück, Osnabrück, Germany; ^4^Institute of Molecular Medicine, Sechenov First Moscow State Medical University, Moscow, Russia

**Keywords:** azoles, drug resistance, *LAM* genes, sterol, stress tolerance, yeast

## Abstract

Lam proteins transport sterols between the membranes of different cellular compartments. In *Saccharomyces cerevisiae,* the *LAM* gene family consists of three pairs of paralogs. Because the function of paralogous genes can be redundant, the phenotypes of only a small number of *LAM* gene deletions have been reported; thus, the role of these genes in yeast physiology is still unclear. Here, we surveyed the phenotypes of double and quadruple deletants of paralogous *LAM2(YSP2)/LAM4* and *LAM1(YSP1)/LAM3(SIP3)* genes that encode proteins localized in the junctions of the plasma membrane and endoplasmic reticulum. The quadruple deletant showed increased sterol content and a strong decrease in ethanol, heat shock and high osmolarity resistance. Surprisingly, the quadruple deletant and *LAM2/LAM4* double deletion strain showed increased tolerance to the azole antifungals clotrimazole and miconazole. This effect was not associated with an increased rate of ABC-transporter substrate efflux. Possibly, increased sterol pool in the *LAM* deletion strains postpones the effect of azoles on cell growth. Alternatively, *LAM* deletions might alleviate the toxic effect of sterols as Lam proteins can transport toxic sterol biosynthesis intermediates into membrane compartments that are sensitive to these compounds. Our findings reveal novel biological roles of *LAM* genes in stress tolerance and suggest that mutations in these genes may confer upregulation of a mechanism that provides resistance to azole antifungals in pathogenic fungi.

## Introduction

Ergosterol is a primary sterol found in the plasma membrane of Ascomycota fungi (Weete et al., 2010). Inhibition of the upstream reactions of ergosterol biosynthesis abrogates cell growth and division (Giaever et al., 2002). While cells can proliferate without the genes *ERG2*, *ERG3*, *ERG4*, *ERG5*, and *ERG6*, which are required for the later steps of the ergosterol biosynthesis pathway (Giaever et al., 2002), deletions of these genes have been shown to decrease yeast fitness under non-optimal conditions (Abe and Hiraki, 2009; Jakubkova et al., 2016; Liu et al., 2017). However, the deletion of *ERG* genes increases the resistance of yeast cells to some stresses, including high osmolarity (Bard et al., 1978) and high tetramethylammonium concentrations (Kodedová and Sychrová, 2015). These effects are linked to hyperpolarization of the plasma membrane in ergosterol-deficient strains (Bard et al., 1978; Welihinda et al., 1994). Moreover, ergosterol plays a major role in the ethanol tolerance of yeast cells (Aguilera et al., 2006), and inhibiting ergosterol biosynthesis at earlier stages of the pathway can increase yeast resistance to some stresses. For instance, partial inhibition of C-14 demethylation of lanosterol (Erg11p) by fluconazole increases the growth rate of yeast cells in the presence of 400 mM NaCl (Montañés et al., 2011); deletion of *ERG* genes increases the growth rate under elevated temperatures of 39.5°C (Liu et al., 2017). Therefore, while being essential for survival in some stressful conditions, high ergosterol content in the plasma membrane can be detrimental in other conditions.

Ergosterol biosynthesis takes place in the endoplasmic reticulum (ER). Ergosterol is subsequently transported to the plasma membrane (PM), by (mostly) non-vesicular mechanisms (Baumann et al., 2005; Alli-Balogun and Levine, 2019; Sokolov et al., 2019). Lam proteins with sterol-binding StART-like domains contribute to ER/PM ergosterol turnover (Gatta et al., 2015). StART domains bind ergosterol and facilitate its transport between membranes (Horenkamp et al., 2018; Tong et al., 2018); therefore, the deletion of *LAM* genes can alter sterol distribution in cells and influence the sterol concentration in PMs.

The phenotypes resulting from mutations in the *LAM* genes remain uncertain. The redundancy of some *LAM* genes further complicates the study. The *Saccharomyces cerevisiae* genome contains three pairs of paralogous *LAM* genes: *LAM1 (YSP1)/LAM3 (SIP3), LAM2 (YSP2)/LAM4,* and *LAM5/LAM6* (Wong and Levine, 2016). While *LAM1/LAM3* and *LAM2/LAM4* paralogs are localized in the contact sites of ER and PM, the localization of *LAM5/LAM6* is not adjacent to the PM (Gatta et al., 2015). It has been shown that Lam6 resides in the mitochondrial/ER and mitochondrial/vacuole contact sites as well as in the nuclear vacuolar junction (Elbaz-Alon et al., 2015). In our study, we focused on Lam1–Lam4 proteins, which appear to have similar intracellular localization.

A single-gene deletion in any of the pairs can be compensated for by the function of the paralog. Nonetheless, single-mutant knockouts of *LAM* genes produce specific phenotypes: (1) the deletion of either *LAM1 (YSP1)* (Pozniakovsky et al., 2005) or *LAM2 (YSP2)* (Sokolov et al., 2006) increases the survival of yeast cells treated with high concentrations of amiodarone. Amiodarone is an antiarrhythmic drug that induces PM hyperpolarization, calcium influx, and acidification of the cytoplasm in yeast cells (Maresova et al., 2009). It also inhibits ABC-transporter-mediated drug efflux in yeasts (Knorre et al., 2009). Importantly, ergosterol biosynthesis mutants are hypersensitive to amiodarone (Gupta et al., 2003). Together, this indicates a strong interaction between *LAM* and *ERG* genes. (2) We also found that the *Δlam2 (ysp2)* strain is resistant to acidification of the cytoplasm induced by acetic acid (Sokolov et al., 2006). (3) The *LAM2* gene confers resistance to antifungal amphotericin B (Gatta et al., 2015) and *LAM3 (SIP3)* to miconazole (François et al., 2009). Given that amphotericin B specifically disrupts ergosterol-enriched membranes, *LAM* mutant strains may carry increased sterol concentrations in the PM. On the other hand, it has been reported that amphotericin B aggregates extracted ergosterol from the yeast PM and that presaturation of amphotericin with ergosterol prevents its antifungal activity (Anderson et al., 2014). Therefore, it is impossible to draw definitive conclusions solely based on amphotericin B sensitivity. To the best of our knowledge, the changes in sterol content observed in yeast *LAM* mutants have not been measured by any direct methods so far. (4) While the deletion of either *LAM2* (*YSP2*) or *LAM3* (*SIP3*) gene increases resistance to quinine, an agent that disturbs membrane integrity, *ERG3* and *ERG6* deletion strains were found to be hypersensitive to quinine (Dos Santos et al., 2014). Nonetheless, the biological role of *LAM* genes is still unclear and the phenotypes of *LAM* mutant strains remain poorly characterized.

In this study, we surveyed the phenotypes of *S. cerevisiae* strains with double deletions of *LAM1/LAM3* and *LAM2/LAM4* paralog pairs. We found that the cells of the quadruple deletant, *Δlam1Δlam2Δlam3Δlam4*, have increased sterol content and decreased resistance to some environmental stresses, such as heat shock, increased salinity, and high ethanol concentrations. During salt stress, we detected a redundancy of gene function, and the *LAM2* gene contributed highly to the major part of the effect on ethanol resistance. Surprisingly, in the *Δlam2Δlam4* strain we observed an increased resistance to azole antifungals. We showed that this effect was not associated with the increased drug efflux by the pleiotropic drug resistance transporters (PDR), although the deletions induced Pdr5 accumulation in the cell PM and the vacuole. We discuss several possible mechanisms which could mediate this increase in azole resistance upon deletion of *LAM* genes.

## Materials and Methods

### Strains, Mediums, and Reagents

Deletion and GFP-fusion strains were obtained by homologous recombination of the PCR product with heterologous selection markers, as listed in Table 1. All strains in this study are derivatives of the *W303* strain. As templates for PCR, the DNA of yeast strains from Euroscarf collection, pUG27 (Gueldener et al., 2002) and pFA6a-His3MX6 (Longtine et al., 1998) plasmids were used. All newly generated strains were verified by PCR with independently designed primers (Table 2) and RT-qPCR (Supplementary Text S1, Table S1, Figure S1). We used standard yeast-rich and synthetic mediums described by Sherman (2002). We obtained yeast extract from BD and D-glucose from Helicon. Clotrimazole, miconazole, nigericin, amphotericin B, propanol, butanol, and FM4–64 were obtained from Thermo Fisher Scientific. Bacto Agar, peptone, NaCl, KCl, and NaN_3_ were obtained from Amresco, and 2-Deoxy-D-glucose was obtained from Chem-Impex Int’l Inc.

**Table 1:** Strains used in this study.

Strain	Genotype	Parental strains and/or references
*W303–1A*	*MATa ade2–101 his3–11 trp1–1 ura3–52 can1–100 leu2–3*	Laboratory of A. Hyman
*Δlam1*	*MATa ade2–101 his3–11 trp1–1 ura3–52 can1–100 leu2–3 Δlam1::HIS3*	*W303–1A*
*Δlam2*	*MATa ade2–101 his3–11 trp1–1 ura3–52 can1–100 leu2–3 Δlam2::TRP1*	Sokolov et al. (2006)
*Δlam3*	*MATa ade2–101 his3–11 trp1–1 ura3–52 can1–100 leu2–3 Δlam3::kanMX4*	*W303–1A*
*Δlam4*	*MATa ade2–101 his3–11 trp1–1 ura3–52 can1–100 leu2–3 Δlam4::HIS3*	*W303–1A*
*Δlam1Δlam3*	*MATa ade2–101 his3–11 trp1–1 ura3–52 can1–100 leu2–3 MATa ade2–101 his3–11 trp1–1 ura3–52 can1–100 leu2–3 Δlam3::kanMX4 Δlam1::HIS3*	*Δlam3*
*Δlam2Δlam4*	*MATa ade2–101 his3–11 trp1–1 ura3–52 can1–100 leu2–3 Δlam2::TRP1 Δlam4::HIS3*	*Δlam2* Sokolov et al. (2006)
*Δlam2Δlam3*	*MATa ade2–101 his3–11 trp1–1 ura3–52 can1–100 leu2–3 MATa ade2–101 his3–11 trp1–1 ura3–52 can1–100 leu2–3 Δlam3::kanMX4 Δlam2::TRP1*	*Δlam3*
*Δlam1Δlam2 Δlam3*	*MATa ade2–101 his3–11 trp1–1 ura3–52 can1–100 leu2–3 MATa ade2–101 his3–11 trp1–1 ura3–52 can1–100 leu2–3 Δlam3::kanMX4 Δlam2::TRP1 Δlam1::NAT*	*Δlam2Δlam3*
*Δlam1Δlam2 Δlam3Δlam4*	*MATa ade2–101 his3–11 trp1–1 ura3–52 can1–100 leu2–3 MATa ade2–101 his3–11 trp1–1 ura3–52 can1–100 leu2–3 Δlam3::kanMX4 Δlam2::TRP1 Δlam1::NAT Δlam4::loxP*	*Δlam1Δlam2Δlam3*
*PDR5-GFP*	*MATa ade2–101 his3–11 trp1–1 ura3–52 can1–100 leu2–3 PDR5-GFP::HIS3*	*W303–1A*
*Δlam1Δlam2 Δlam3Δlam4 PDR5-GFP*	*MATa ade2–101 his3–11 trp1–1 ura3–52 can1–100 leu2–3 MATa ade2–101 his3–11 trp1–1 ura3–52 can1–100 leu2–3 Δlam3::kanMX4 Δlam2::TRP1 Δlam1::NAT Δlam4::loxP PDR5-GFP::HIS3*	*Δlam1Δlam2 Δlam3Δlam4*
*PGAL-ERG9*	*MATa ade2–101 his3–11 trp1–1 ura3–52 can1–100 leu2–3 PGAL-ERG9::HIS3*	*W303–1A*
*UPC2–1*	*MATa UPC2–1 ura3–1 his3–11,-15 leu2–3,-112 trp1–1*	

**Table 2:** Primers used in this study.

LAM1-F1 5′-ttcaagtttttcacttctatagctttggtattggtcattgtagaacaattttataCGGATCCCCGGGTTAATTAA
LAM1-R1 5′-ttagacaagacggggtccttctgattattgaagagtagacattctggggcactatGAATTCGAGCTCGTTTAAAC
LAM1-R test 5′-GCGCGTAAGAATCACCTGAT
Test primer 5′-GTTTAAACGAGCTCGAATTC
LAM2-F 5′-CGTTAGTCCACCATAACCAA
LAM2-R 5′-CCAGATATAGATGCTATATG
LAM2-F test 5′-CGTTTAATATCGTCAACGAC
LAM2-R test 5′-GATATGCGAGCTCTTCATCT
LAM3-F 5′-GAAGACGCTATCACTTTTAC
LAM3-R 5′-CTGACAAATTTAACGTAATCC
LAM3-F test 5′-GTAGACATTTCTGAGGCATT
kanMX160 5′-GACAGTCACATCATGCCCCT
LAM4-F pUG 5′-tactgtgtgtggttcttactctccatggatagtgttgaaaatatacagtaCAGCTGAAGCTTCGTACGC
LAM4-R pUG 5′-actagatacaattactaaataatacaaacagaatatataaaatgctattaGCATAGGCCACTAGTGGATCTG
LAM4 test 5′-TACGCTGATATGAAAATGCT
pUG F test 5′-GCGTACGAAGCTTCAGCTG
Pdr5-GFP-Sh-F 5′-ACGGTAAACTCTCCAAGAAA
Pdr5-GFP-Sh-R 5′-ACGCACCTATATGTAGTGAT

### Quantification of Total Cellular Ergosterol Content

Cells were grown overnight in 20 ml of YPD in 100-ml flasks upto the stationary phase and then centrifuged and resuspended in 20 ml of water. Fifteen milliliters of the cell suspension were transferred onto a drying filter and dried to a constant weight at 95°C. Thus, the dry weight of 15 ml of cell suspension was determined. Ergosterol content in 1 ml of cell suspension was determined as described in by Arthington-Skaggs et al. (1999) with modifications. One milliliter of the cells was concentrated by brief centrifugation and resuspended in 10 μl of water. The cells were then resuspended in 500 μl of a 25% solution of KOH in ethanol (1 g of KOH was dissolved in 1.4 ml of water and adjusted to 4 ml with ethanol) for 1 h at 85°C. The solution was cooled to room temperature, and sterol was extracted into a mixture of 500 μl of heptane with 100 μl of water by vortexing for 3 min. The absorption spectrum of sterol was recorded using spectrophotometer (SpectrostarNANO) in the Microplate UV–VIS, 96/F (Eppendorf) in 200 μl of sample.

### Filipin Staining

Filipin staining was performed according to the method described by Grossmann et al. (2007). Living cells were washed in 50 mM potassium phosphate buffer, pH 5.5, diluted to *A*_600_ = 0.3, stained with 5 μg/ml filipin (Sigma) for 5 min, washed again in the same buffer, concentrated by brief centrifugation, and analyzed using a fluorescent microscope with a U-MNU2 filter set (*λ*_exitation_ = 360–370 nm; dichroic mirror *λ* = 400 nm; *λ*_emission_ > 420 nm). Because filipin is very prone to photobleaching, we started fluorescence exposure of each field of view simultaneously with accumulation of the camera signal.

### Colony Growth on 0.3% Agar

Cells were grown overnight to exponential phase, then diluted to an optical density of OD_550_ = 0.2 (Thermo Genesys2). Two microliters of cell suspension were transferred onto 0.3% agar YNB complete plates. Colony morphology was analyzed on the fifth day of growth.

### Growth Kinetics

Exponentially growing cells were diluted to an optical density of OD_550_ = 0.2 and inoculated into a 48-well plate (Greiner). Plates were incubated in a spectrophotometer (SpectrostarNANO) with the following settings: orbital shaking at 500 rpm for 2 min at 30°C before measurements; measurements were performed at 5-min intervals. To quantify the results in non-stressful conditions, we compared the growth rates (μ) between the control and mutant strains. In stressful conditions, we compared the increase in OD between the first and tenth hour of growth. We excluded first-hour measurements from the analysis due to artifacts in the first measurements, which could be attributed to fogging of the plate lid.

### Survival Experiments

Cells were grown overnight to the logarithmic growth phase, then diluted to OD_550_ = 0.2. We measured the survival of cells as the number of colony forming units (CFU) in the mutant strain normalized to unstressed cells. We evaluated the number of colonies after 24 h of growth at 30°C on solid YPD medium. Hundred percent represented the number of CFU at the moment of stress exposure (*t* = 0). Cells were subjected to either 12% (v/v) ethanol for 1 h, 2.3 M NaCl for 1 h, heat shock (47°C for 30 min), or freezing (−20°C for 18 h).

### Flow Cytometry

Fluorescence of GFP was assessed with a Beckman Coulter FC 500 flow cytometer using an excitation wavelength of 488 nm on the emission filter (525/40 nm). The accumulation of Nile red and rhodamine 6G was measured with an emission filter (575/20 nm). At least 30,000 events were analyzed in each experiment. We calculated the average fluorescence intensity in populations for each separate-day biological replicate. Cells with GFP expression were grown overnight in solid YPD medium and then resuspended to a density of 2 × 10^5^ cells/ml in the same medium. Fluorescence was assessed after 1 h of preincubation with clotrimazole or solvent at 30°C.

### Rhodamine 6G and Nile Red Efflux

Cells were grown overnight on solid YPD medium, resuspended to a density of 2 × 10^5^ cells/ml in phosphate-buffered saline (PBS, Gibco), and supplemented with 5 mM 2-Deoxy-D-glucose and 10 mM NaN_3_. After 2 h of incubation with Nile red (3.5 μM) or rhodamine 6G (400 nM) at 30°C, cells were washed twice with PBS and resuspended in PBS with 0.1% glucose. The amount of Nile red and rhodamine 6G in the yeast cells was measured by flow cytometry.

### Fluorescent Microscopy

To study the accumulation of GFP, yeast cells were visualized using an Olympus BX41 microscope with the U-MNIBA3 filter (excitation wavelength 470–495 nm; beam splitter filter 505 nm; emission 510–550 nm) for GFP, and the U-MNG2 filter (excitation wavelength 530–550 nm, beam splitter filter 570 nm; emission >590 nm) for FM4–64. Photographs were taken with a DP30BW CCD camera. All results were reproduced in at least three biological replicates. To visualize vacuolar membranes, yeast cells were stained with 0.8 μM FM4–64 (Thermo Fisher Scientific) at 30°C for 1 h in YPD with clotrimazole or solvent, then washed twice with PBS.

### Data Visualization and Analysis

We compared different strains and conditions using the Wilcoxon rank-sum test or Wilcoxon signed-rank test with the Bonferroni adjustment for multiple comparisons. To visualize the data, we used *R* software (R Core Team, 2014) and provided individual data points when possible.

## Results

**Figure 1:**
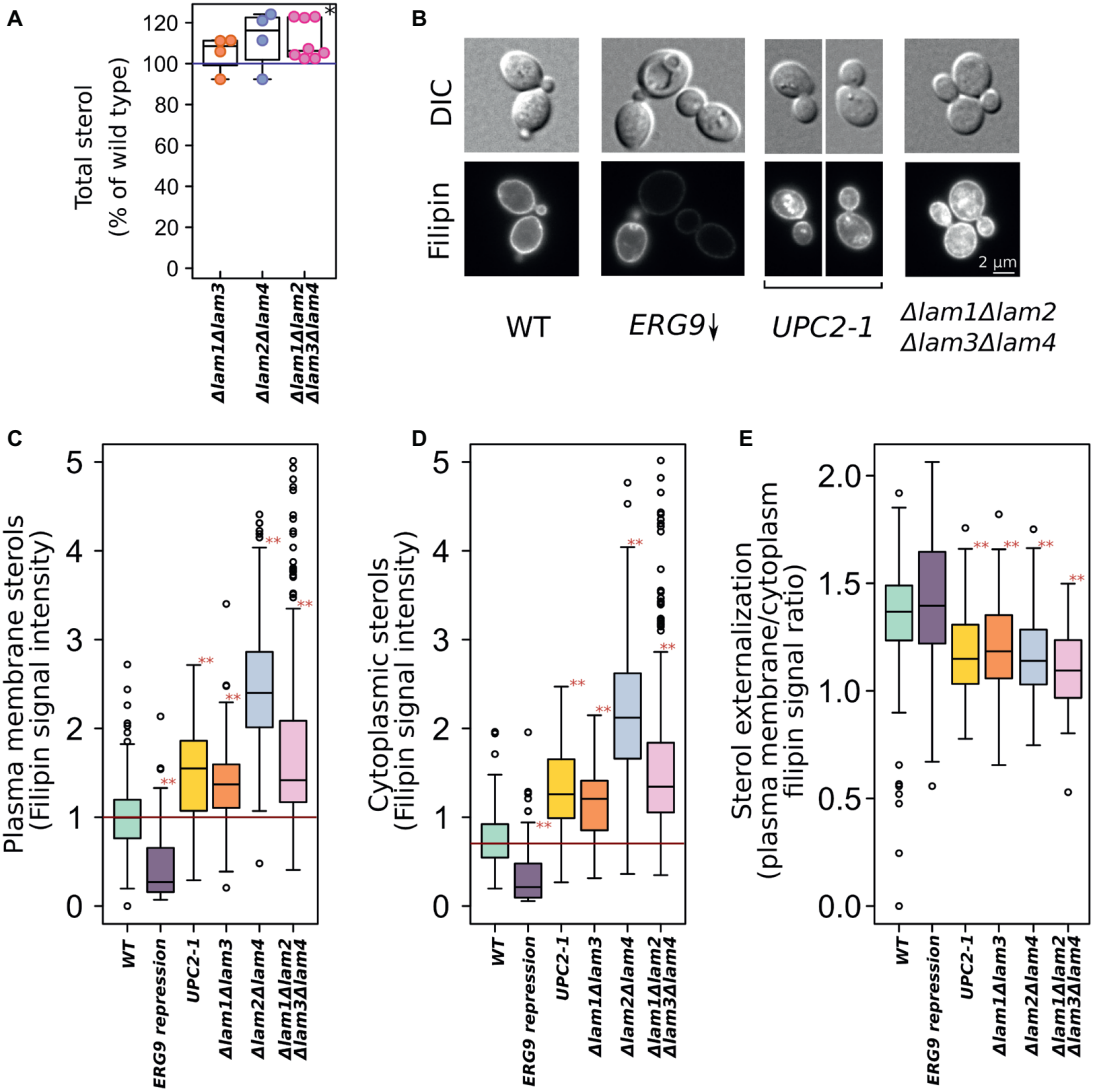
Increased sterol concentrations in the strains with deleted *LAM* genes. **(A)** Total sterol content was measured *via* light absorbance at *λ* = 282 nm of the wild-type and deletion strain cells. Plasma membrane ergosterol levels were assessed *via* fluorescent microscopy using filipin, a sterol-binding fluorescent dye. **(B)** Representative photographs of the wild-type and mutant yeast cells stained with filipin. Yeast cells were grown in YPD plates and then analyzed using a fluorescent microscope. This condition ensured repression of P_GAL_-ERG9 gene. Quantification of filipin plasma membrane **(C)** and cytoplasmic **(D)** staining for strains from B as well for mutant strains with double deletions. **(E)** Ratio of PM/inner compartments filipin staining. ^*^p < 0.01 for comparisons with the *WT*, paired Wilcoxon signed-rank test; ^**^p < 10^−7^ for comparisons with the *WT*, paired Wilcoxon signed-rank test with Bonferroni adjustment.

We generated a set of *S. cerevisiae* strains with deleted pairs of *LAM* paralogs (see Table 1). *LAM1/LAM3* and *LAM2/LAM4* genes represent the paralog pairs that arose as a result of whole-genome duplication and were preserved in the *S. cerevisiae* genome during evolution. The quadruple deletion strain showed increased total sterol concentration (Figure 1A). To determine the intracellular distribution of sterol, we stained yeast cells with filipin, a sterol-sensitive fluorescent dye. Filipin interacts with sterol but not with esterified sterol [see Wilhelm et al., 2019]. To validate the usage of filipin, we took a strain with conditionally regulated *ERG9* gene and that with a dominant gain of function mutation in sterol-sensitive transcription factor *UPC2–1* and demonstrated that 24 h of preincubation of P_GAL_-*ERG9* strain in YPD plates (the condition of P_GAL_-*ERG9* repression) significantly reduced filipin staining of yeast cells (Figure 1B). Furthermore, the *UPC2-1* allele increased filipin staining in both the cytoplasm and PM (Figures 1B–D). Therefore, we concluded that in our experimental system, filipin can be used to visualize sterol. We quantified filipin fluorescence in the PMs (Figure 1C) and cytosol (Figure 1D) of the wild-type and *LAM* mutant cells. Deletion of either *LAM* gene pairs increased filipin staining, suggesting an increase in sterol accumulation. Surprisingly, in the *UPC2-1* and *LAM* double and quadruple deletion strains, we detected cytoplasmic staining (Figure 1E). It appeared that despite an increase in the plasma membrane sterol levels, the *UPC2-1* and *LAM* deletants showed redistribution of non-esterified sterol from PM into the inner cellular compartments.

**Figure 2:**
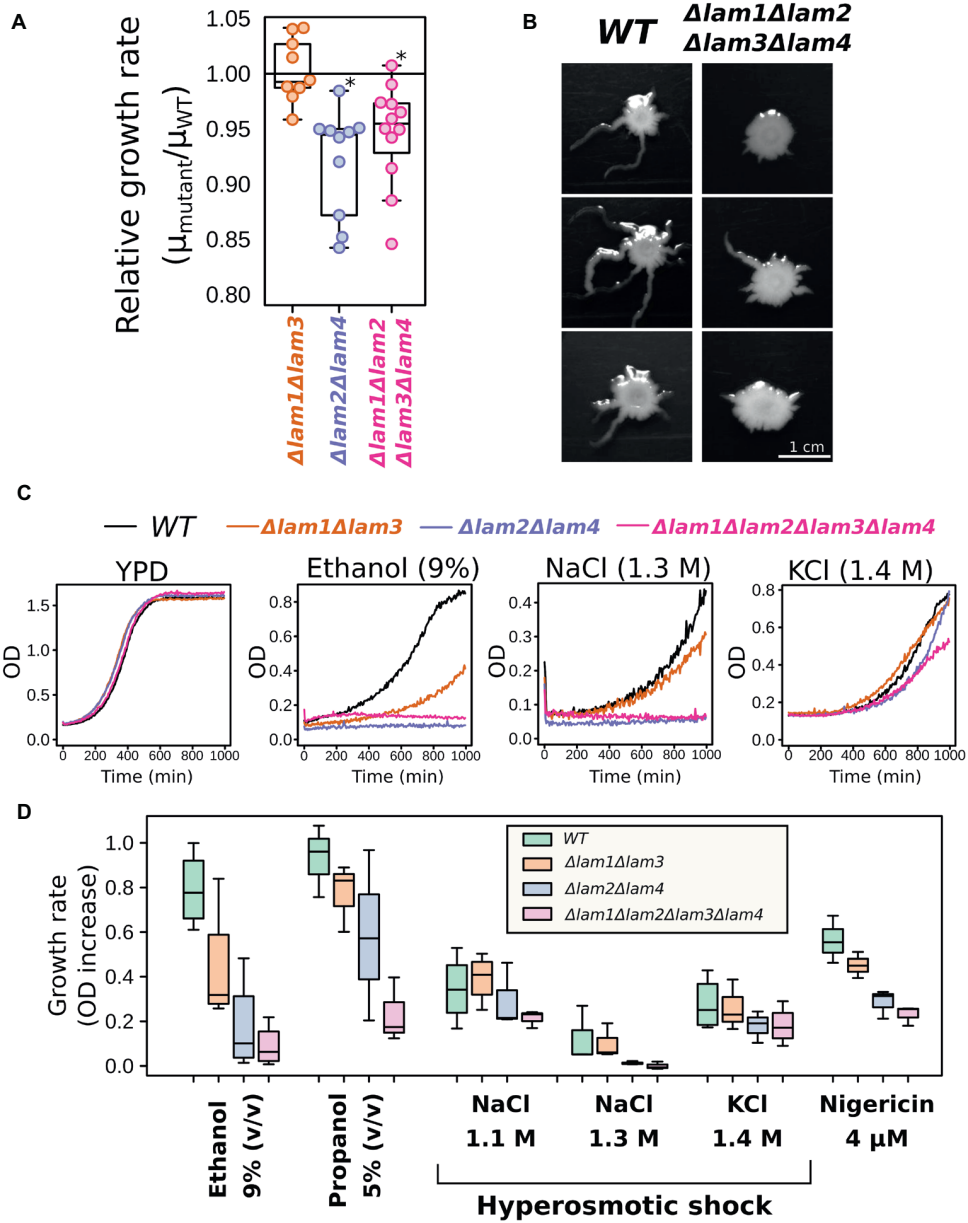
Growth rate phenotypes of *LAM1-4* deletion mutants. **(A)** Deletion of *LAM2* and *LAM4* paralogs decreases relative growth rate in YPD; ^*^p < 0.05, Wilcoxon signed-rank test. **(B)** Yeast colonies after 5 days of growth in low-agar solid medium, representative photographs (*n* = 3). **(C,D)** *LAM2* and *LAM4* significantly contribute to growth rate in stressful conditions. **(C)** Representative growth curves. **(D)** Quantification of the results. (*n* = 3–4).

When analyzing growth kinetics in rich medium with glucose as carbon source (YPD), we detected a small decrease in growth rate for *Δlam1Δlam2Δlam3Δlam4* and *Δlam2Δlam4* mutant strains (Figure 2A). Moreover, although *LAM* deletions conferred no effect of *LAM* deletions on colony size in solid YPD medium, the quadruple mutant strain showed decreased ability to invade low-agar media (Figure 2B). Next, for the preliminary screening of phenotypes, we assessed the effects of alcohols (ethanol and propanol) and hyperosmolarity conditions. The resistance to these stresses depends on the PM sterol concentrations, according to previously published reports (see section “Introduction”). We found that all tested conditions, with the exception of high KCl concentration, inhibited the growth of the mutants that lacked *LAM2* and *LAM4*, while the effect of the double deletion of *LAM1* and *LAM3* was less pronounced in all tested cases (Figures 2C,D).

**Figure 3:**
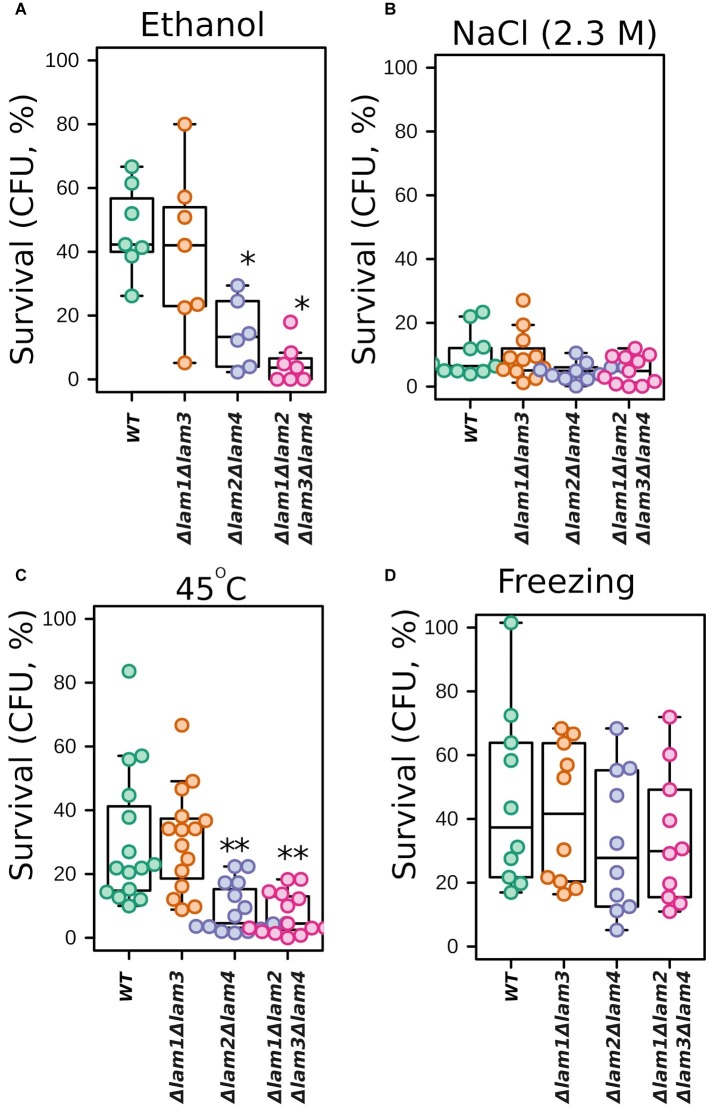
Survival of *LAM* deletion mutants exposed to stresses. The survival was measured as the number of colony forming units (CFU) in the mutant strain normalized to unstressed cells. 100% represents the number of CFU at the beginning of stress exposure. Cells were subjected to 12% v/v ethanol for 1 h **(A)**; 2.3 M NaCl for 1 h **(B)**; heat shock (47°C for 30 min) **(C)**; or freezing (−20°C for 18 h) **(D)**. Experiments conducted in YPD with cells grown to exponential phase. ^*^p < 0.05, ^**^p < 0.005 for comparisons with *WT*, unpaired Wilcoxon-Mann-Whitney test.

We tested the survival of the double and quadruple *LAM* mutants under selected extreme environmental stresses: high osmolarity, high ethanol concentration, heat shock, and freezing. In line with growth experiments, we observed decreased survival of *Δlam1Δlam2Δlam3Δlam4* and *Δlam2Δlam4* cells in the presence of high concentrations of ethanol and under lethal heat shock (Figures 3A–D). However, both strains showed no significant change in survival after high hyperosmolarity stress or freezing (Figures 3B,D).

**Figure 4:**
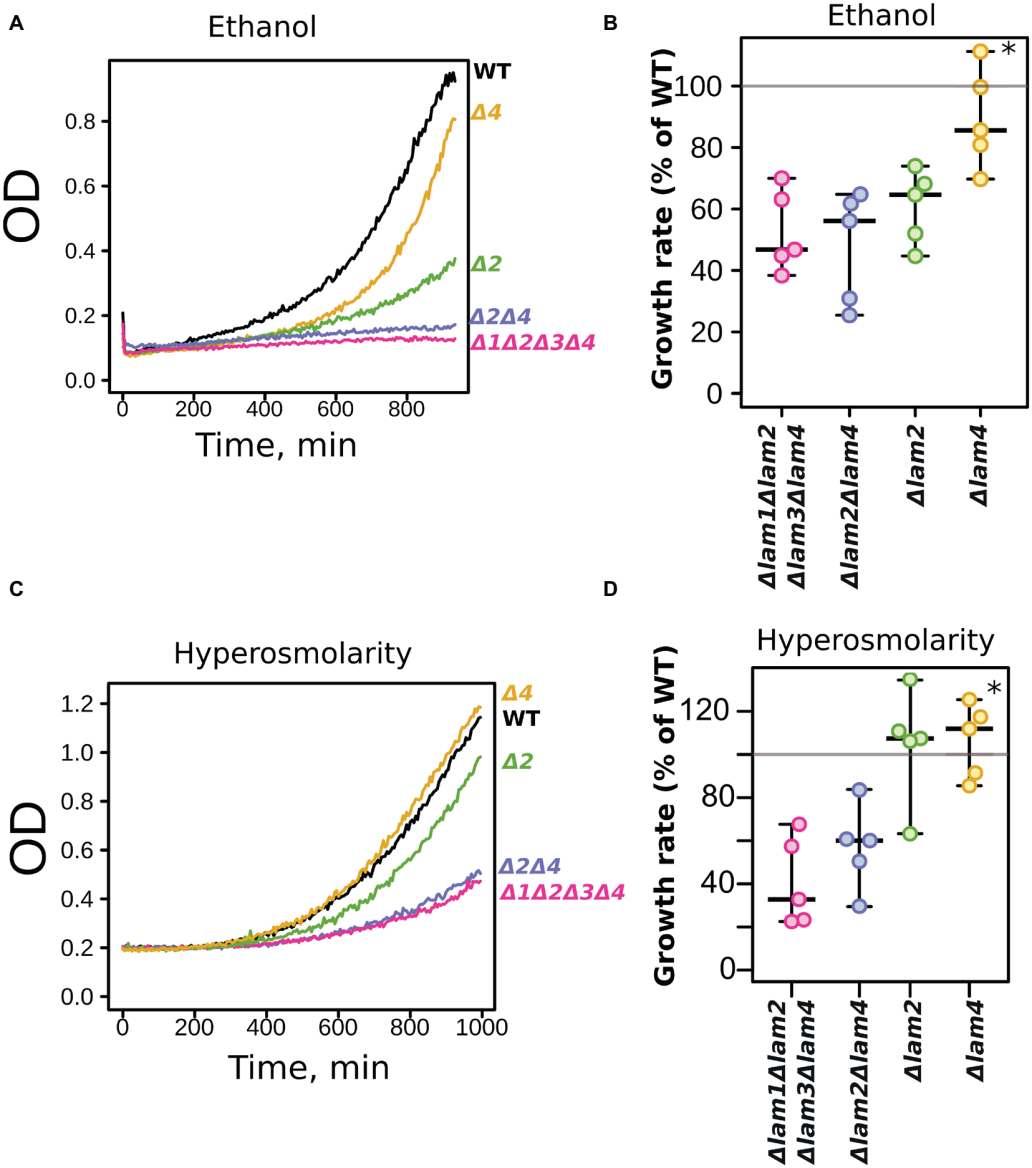
Relative contribution of *LAM2* and *LAM4* genes to ethanol (9% v/v) **(A,B)** and hyperosmolarity tolerance (1.1 M NaCl) **(B,C)**. Representative growth curves **(A,C)** and quantification of OD during 9 h of growth **(B,D)**. 100% represents the growth of the *WT* (parental) strain in the same conditions. ^*^p < 0.02 for comparisons with *Δlam2Δlam4,* unpaired Mann-Whitney test with Bonferroni adjustment.

The *Δlam2Δlam4* knockout strain showed more pronounced phenotypes than the *Δlam1Δlam3* strain. Thus, we decided to test the relative contributions of *LAM2* and *LAM4* to ethanol/high salinity tolerance. We compared the growth kinetics of *Δlam2Δlam4* with two single-gene knockout strains, *Δlam2* and *Δlam4* (Figure 4). We found that the effect on the growth rate in the presence of 9% ethanol is determined mainly by the *LAM2* gene (Figures 4A,B). Meanwhile, the functions of *LAM2* and *LAM4* appeared to be redundant for the high osmolarity growth phenotype (Figures 4C,D).

**Figure 5:**
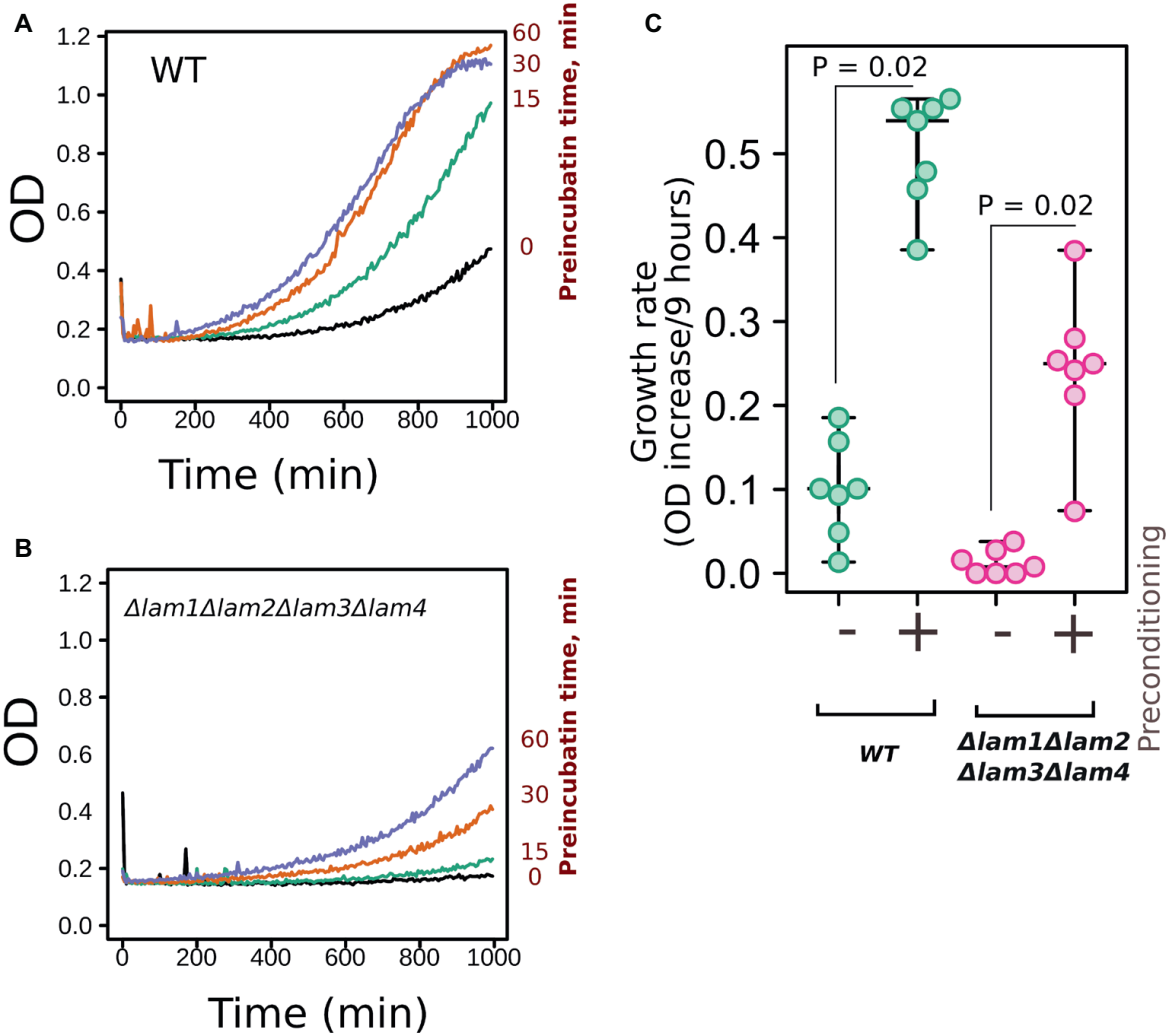
Deletion of *LAM* genes did not prevent yeast preadaptation to high osmolarity. **(A,B)** Yeast cells of control (*WT*) or quadruple mutant *Δlam1Δlam2Δlam3Δlam4* strains were incubated with 0.4 M of NaCl for different time intervals (indicated in the figure) and then the concentration of NaCl was increased to 1.1 M. We measured yeast growth kinetics starting from this point. **(C)** Quantification of the results presented in Figures 5A,B. Increases in OD during first 9 h of growth are indicated. Experiments were conducted in YPD with yeast cultures in exponential growth phase. Values of *p* were calculated according to Wilcoxon signed-rank test.

Yeast response to high osmolarity stress relies on multiple proteins and systems (Hohmann, 2015). These systems include PM osmosensor proteins, which sense the changes in membrane properties and transduce the signal to the high osmolarity glycerol pathway (Tatebayashi et al., 2015). Thus, it is possible that the deletion of *LAM* genes interferes with the ability of yeast cells to adapt to increased osmolarity. In this case, the deletion of *LAM* genes would have prevented the preadaptation response to increased NaCl concentration in the incubation medium. To test this theory, we incubated yeast cells in the presence of 0.4 M NaCl and then increased the concentration to 1.1 М. Such preadaptation increased the growth rate of both the wild-type strain and the quadruple deletion strain, *Δlam1Δlam2Δlam3Δlam4*, in the presence of 1.1 M NaCl (Figure 5). Therefore, the absence of Lam proteins in PM-ER junctions did not prevent the ability of yeast cells to induce a hyperosmolarity response.

**Figure 6:**
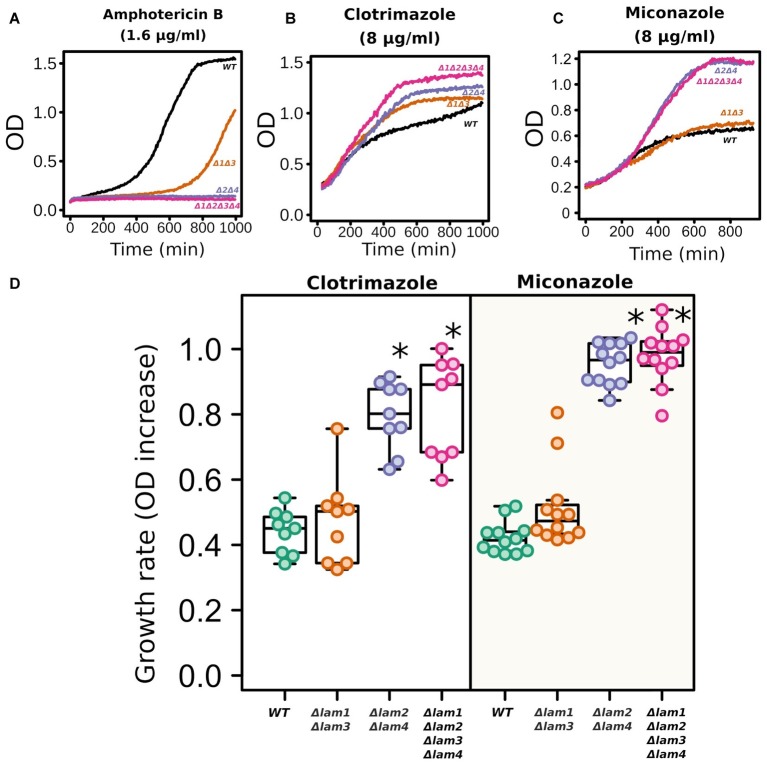
*LAM* deletion strains are resistant to azole antifungals. Amphotericin B (1.6 μg/ml), clotrimazole (8 μg/ml), and miconazole (8 μg/ml). Representative growth curves **(A–C)** and quantification of the results **(D)**. For quantification, we used the increase in OD_λ550_ during 9 h of growth. ^*^p < 0.001 for comparisons with *WT* strain, unpaired Mann–Whitney test with Bonferroni adjustment.

While the deletion of *LAM* genes decreased the resistance to environmental stresses, it increased the resistance to azole antifungals (Figure 6). In line with the results published by Gatta et al. (2015), *LAM* genes provided resistance to polyene antimycotic amphotericin B (Figure 6A). Amphotericin targets ergosterol-rich membranes: it absorbs ergosterol from the PM and consequently disrupts its barrier function (Anderson et al., 2014). Surprisingly, the addition of an azole antifungal (clotrimazole or miconazole) significantly decreased the survival of the wild-type cells but not *LAM* mutant cells (Figures 6B–D).

**Figure 7:**
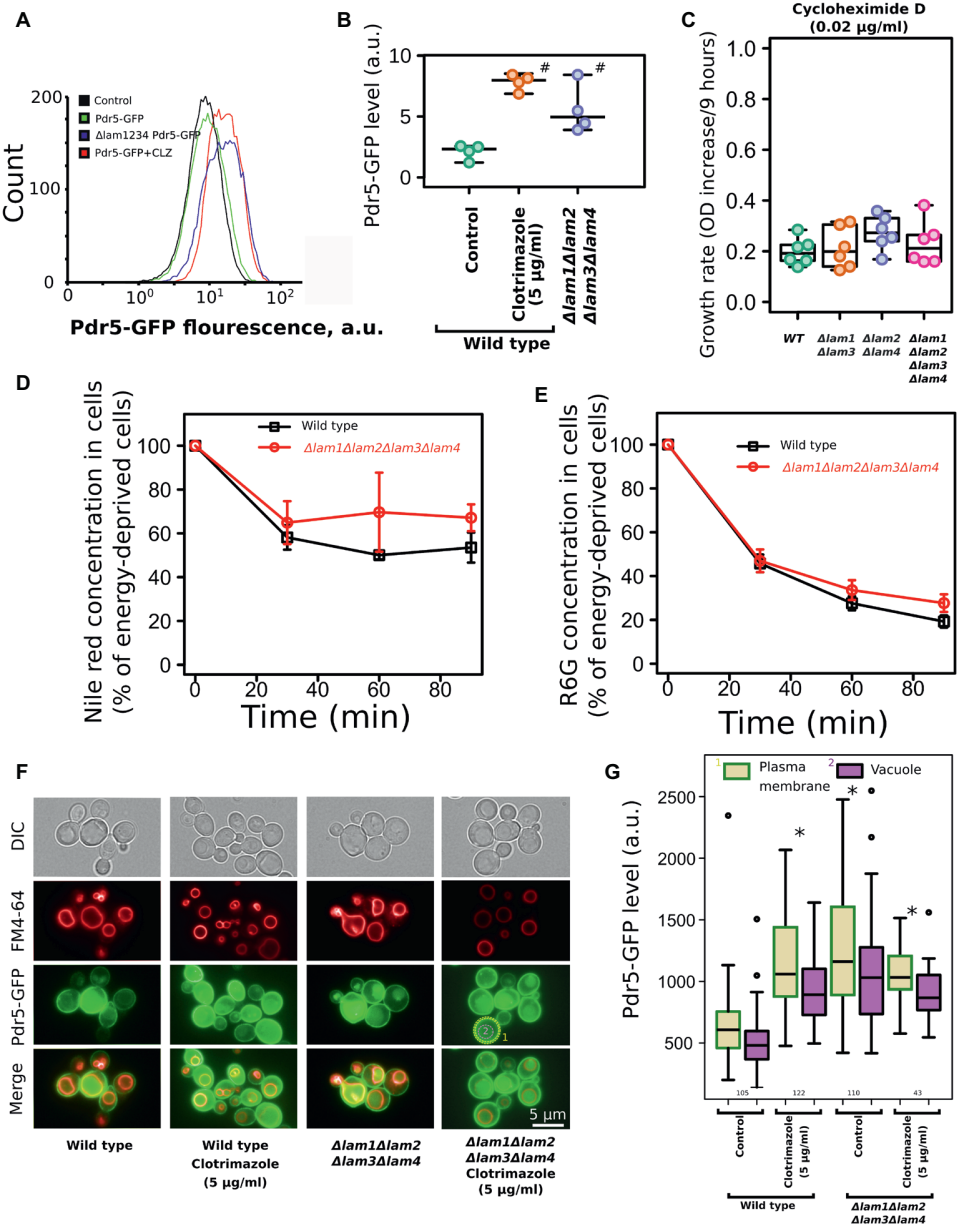
Deletion of *LAM* genes increases the level of ABC-transporter Pdr5-GFP but does not increase the efflux rate of ABC-transporter substrates. **(A)** Flow cytometry analysis of Pdr5-GFP wild-type and *Δlam1Δlam2Δlam3Δlam4* strains. As a positive control, to induce Pdr5-GFP accumulation, we treated the wild-type strain with clotrimazole (CLZ, 5 μg/ml); a control strain lacking the *PDR5-GFP* gene was used as a negative control. **(B)** Quantification of (A), ^#^corresponds to *p* = 0.057, for comparisons with *WT* strain, unpaired Mann–Whitney test with Bonferroni adjustment. **(C)** The increase of OD during first 9 h of growth of the mutant strain with cycloheximide D, a protein synthesis inhibitor and a substrate of PDR transporters (0.02 μg/ml). Experiments were conducted in YPD with yeast cultures in exponential growth phase. **(D,E)** The dynamics of pumping the ABC-transporter substrates Nile red **(D)** and rhodamine 6G **(E)** out of wild-type and *Δlam1Δlam2Δlam3Δlam4* cells (*n* = 3). **(F)** Photograph of yeast cells expressing Pdr5-GFP stained with FM4–64. The average signal intensity of plasma membrane (outlined by yellow dashed lines) was compared to the average signal in vacuole (red line). **(G)** Quantification of the plasma membrane/vacuolar signal intensity ratio. Numbers of analyzed cells pooled from two biological replicates are indicated below the boxplots. ^*^p < 10^−8^ for comparisons to PM Pdr5-GFP level in control cells, unpaired Mann–Whitney test with Bonferroni adjustment.

One of the key mechanisms that provide azole tolerance is the upregulation of PDR transporters that extrude xenobiotic compounds from cells (Prasad et al., 2015). An increase in azole resistance can be attributed to the upregulation or activation of these transporter proteins, which is triggered by sterol deficiency (Vu et al., 2019). To test whether azole tolerance in the *LAM* quadruple mutant is mediated by PDR transporters, we measured the accumulation of PDR substrates and the level of main ABC-transporter in the wild-type strain and in the *Δlam1Δlam2Δlam3Δlam4* strain. First, we measured the level of Pdr5-GFP by flow cytometry. We produced the mutant strain by fusing GFP to the genomic copy of the *PDR5* gene. Pdr5 is one of the major pleiotropic drug resistance ABC-transporters in *S. cerevisiae*. We found that the level of Pdr5-GFP was increased in the *Δlam1Δlam2Δlam3Δlam4* strain (Figures 7A,B). We used clotrimazole as a positive control with increased Pdr5 levels (see Galkina et al. (2018)). However, we did not detect an increase in PDR activity in the *Δlam1Δlam2Δlam3Δlam4* mutant strain. First, there was no pronounced difference in the resistance to cycloheximide between the *LAM* mutant strains. Cycloheximide is a protein synthesis inhibitor and a substrate of the Pdr5 transporter (Leppert et al., 1990). Surprisingly, despite an increase in Pdr5 concentration in the *LAM* quadruple mutant strain, we did not detect a pronounced difference between *LAM* knockout strains (Figure 7C). Moreover, we did not detect an increase in efflux of the PDR fluorescent substrates Nile red (Figure 7D) and rhodamine 6G (Figure 7E) in the *LAM* quadruple mutant.

Alteration of membrane sterols could affect the targeting of membrane proteins to the PM. For example, in *Candida albicans* deletion of the ergosterol biosynthesis pathway genes *Δerg24*, *Δerg6,* and *Δerg4* induces mistargeting of СaCdr1, a Pdr5 ortholog, to vacuoles instead of the PM (Pasrija et al., 2008). To test if the deletion of *LAM* genes changes the localization of Pdr5, we analyzed the intracellular localization of Pdr5-GFP in wild-type and mutant strains. We found that quadruple deletion of *LAM* genes did not induce intracellular redistribution of Pdr5-GFP and that in *Δlam1Δlam2Δlam3Δlam4* strain, Pdr5-GFP was predominantly localized in the PM (Supplementary Figure S2). In agreement with the flow cytometry data, we detected increased concentration of Pdr5-GFP in clotrimazole-treated and *Δlam1Δlam2Δlam3Δlam4* cells. Moreover, the signal from GFP increased in the PM as well as in the vacuole (Figures 7F,G). However, we did not detect an additional increase in PM Pdr5-GFP levels upon the treatment of yeast cells with clotrimazole (Figures 7F,G). These results suggest that increase in azole tolerance in the *Δlam1Δlam2Δlam3Δlam4* mutant strain is associated with increased Pdr5 levels, but the quadruple deletion does not provide a pronounced increase in drug efflux activity.

## Discussion

In mammalian cells, membrane-bound sterol-transporting proteins internalize high-density lipoprotein cholesterol to intracellular compartments (Sandhu et al., 2018). However, the biological role of these proteins is still unclear due to the absence of phenotypes for single-gene knockdowns. The genome of *S. cerevisiae* contains three pairs of paralogous membrane-anchored sterol-transporter genes (*LAM* genes). These paralogous genes arose because of whole-genome duplication. In this study, we produced a quadruple knockout strain, *Δlam1Δlam2Δlam3Δlam4* (Table 1), that lacks all four genes that encode the PM-ER-tethered Lam proteins. Lam5p and Lam6p are localized in ER-mitochondria and ER-vacuolar junctions and were not analyzed in our study.

In the quadruple deletion strain, we assessed total sterol content *via* spectrophotometry and analyzed the intracellular distribution of non-esterified sterols using the sterol-binding fluorescent dye filipin. We found that quadruple deletion increased total sterol content by a factor of approximately 1.11 (Figure 1A). Because Lam2 protein mediates retrograde sterol transport from the PM to the inner compartments (see Gatta et al., 2015), the deletion of *LAM1-4* genes may increase the PM sterol concentration relative to that in the inner compartments. Meanwhile, we detected sterol redistribution from the PM to the cytoplasm. While the wild-type parental strain showed strong PM staining and a clear cytoplasm, in *LAM* mutants, we detected cytoplasmic staining (Figure 1B), reflecting either increased ER or lipid droplets sterol levels or decreased sterol esterification levels in the inner compartments. Strikingly, the filipin-staining phenotype of *LAM* deletions was similar to that of the gain of function dominant mutation*, UPC2-1,* of the sterol-sensing transcription factor (Crowley et al., 1998). We speculate that the deletion of *LAM* genes decreases the sterol available for Upc2-mediated sensing, resulting in its activation. We are currently investigating this possibility. Nonetheless, filipin staining revealed increased sterol concentrations in the PM (Figures 1B,C).

We found that the double and quadruple mutants show slightly decreased growth rates under standard fermentable conditions (Figure 2A). High growth rate under optimal conditions is usually antagonistic to stress resistance due to reallocation of resources between proliferation and stress tolerance. Indeed, many strains with low growth rates show increased stress tolerance (Zakrzewska et al., 2011). Meanwhile, in our study, the quadruple *LAM* deletion mutant was sensitive to certain environmental stresses: high salinity, alcohols (Figures 2, 3), and heat shock (Figure 3). It is known that ergosterol levels determine a cell’s resistance to these stresses. For example, sterol deficiency in yeast cells decreases ethanol tolerance and heat shock tolerance, but this effect is not due to changes in trehalose or heat shock-protein levels (Swan and Watson, 1998). Moreover, the stability of the PM depends on its sterol composition: a high sterol concentration makes membranes more resistant to different surfactants (Apel-Paz et al., 2005). Therefore, we suggest that the phenotypes of the *Δlam1Δlam2Δlam3Δlam4* strain are explained directly by altered PM sterol content rather than by indirect effect on stress-response protein machinery. It is also possible that the deletion of *LAM* genes prevents activation of cell stress-response signaling and activation of the generalized stress-response pathway. However, in our experiments, the deletion strain retained the ability to increase NaCl tolerance in response to preconditioning to moderate salinity (Figure 4).

The conservation of four paralogous genes (*LAM1–LAM4*) in fungi, which underwent whole-genome duplication, suggests that, despite the redundancy in function, each Lam protein has its own unique function. Our study revealed that *LAM2* (*YSP2*) plays a major role in ethanol tolerance: the effect of *LAM2* deletion was the same order of magnitude as the effect of quadruple deletion (Figures 4A,B). At the same time, a copy of *LAM4* gene was sufficient to confer growth in increased salinity (Figures 4C,D). This differential response is unlikely to be a result of different activities or concentrations of Lam2p and Lam4p. Indeed, according to available global proteomic analysis, Lam2p (Ysp2p) is approximately twice as abundant in yeast cells compared to Lam4p (Ho et al., 2018). However, it is also possible that the low-abundance protein, Lam4p, transports minor sterol biosynthesis intermediates, while Lam2p is responsible for ergosterol transport or has wider substrate specificity. The substrate specificity of Lam proteins is still unknown; however sterol-binding pockets in their StART-like domains are strongly conserved within the paralogous pair Lam2/Lam4 (Horenkamp et al., 2018).

Deletion of *LAM* genes did not always decrease cell fitness under the conditions that we tested. The absence of *LAM2* and *LAM4* increased the cell’s tolerance to azoles (Figure 6). We considered four possible mechanisms of this effect:

1. The deletion of *LAM* genes can upregulate PDR PM transporters. These transporters extrude azoles from the cytoplasm at the cost of ATP hydrolysis (Prasad and Goffeau, 2012). Indeed, we detected an increase in Pdr5-GFP in yeast PMs in the quadruple *LAM* mutant (Figure 7). However, despite the increased concentration of the protein, we did not observe an increase in ABC-transporter substrate efflux rate in this strain (Figure 7). This discrepancy suggests that the absence of *LAM* genes increases Pdr5 concentration and simultaneously decreases the activity of this protein. Indeed, the activity of PDR ABC-transporters is sensitive to the sterol composition of the PM (Kodedová and Sychrová, 2015). We suggest that a decrease in ABC-transporter activity could induce Pdr5 accumulation as a compensatory mechanism.
2. Azole-induced ergosterol depletion (Watson et al., 1989) can also contribute to the cytostatic effect on yeasts. Therefore, a mutation that increases sterol concentration in cells may make additional divisions by cells exposed to azoles possible. If ER-PM Lam proteins facilitate retrograde ergosterol traffic from the PM to the ER (Gatta et al., 2015), then their deletion can increase PM ergosterol concentration. Indeed, deletion of *LAM* genes increased total and PM sterol content (Figure 1), supporting this explanation of azole-resistant phenotype.
3. Azole antifungals inhibit lanosterol demethylase Erg11p, which catalyzes the reaction of lanosterol demethylation in the ergosterol biosynthesis pathway (Sant et al., 2016). The inhibition of Erg11p decreases the PM sterol level and induces the accumulation of toxic sterol intermediates (Kelly et al., 1995; Martel et al., 2010). Here, we speculate that Lam proteins can contribute to the transport of these intermediates to a compartment sensitive to these intermediates, i.e., the PM. Indeed, ergosterol is synthesized in the ER and only then redistributed to other cellular membranes (Zweytick et al., 2000).
4. Deletion of *LAM* genes could have affected the lipid composition of the PM. These changes can theoretically selectively increase PM permeability to one compound, while decrease it to others. However, although we cannot rule out this possibility, it is unlikely that the deletion of *LAM* genes decreases permeability specifically to azoles but not the other compounds tested in our study (cycloheximide, nigericin, Nile red, and rhodamine 6G). Our data did not allow us to discriminate between the above-discussed mechanisms. Moreover, being non-exclusive, these mechanisms may collectively contribute to the observed effects.

To summarize, we have characterized the phenotype of a strain of *S. cerevisiae* that had four *LAM* genes deleted that encode membrane-anchored sterol transporters localized in PM-ER junctions. We found that the deletions increased yeast sensitivity to environmental stresses but alleviated azole sensitivity. In all tested cases, the effect of *LAM2* and *LAM4* deletion was more pronounced than the effect of *LAM1* and *LAM3* deletion. Altered sensitivity of *LAM* deletants to azole antifungals suggests that the mutations in these genes can be an additional route for evolution of azole tolerance in pathogenic fungi.

## Data Availability Statement

All datasets generated for this study are included in the article/Supplementary Material.

## Author Contributions

SS, FS, and DK designed the study. SS and FS acquired funding. SS, NT, and AS generated mutant strains. SS, NT, ES, MV, and KG measured growth rate kinetics and cell survival in stressful conditions. AS, SS, and DK performed microscopy experiments. KG performed flow cytometry and fluorescent substrate efflux experiments. SS, KG, AS, and DK analyzed the data. DK prepared the illustrations and drafted the manuscript. All authors contributed to manuscript editing and approved the final version.

### Conflict of Interest

The authors declare that the research was conducted in the absence of any commercial or financial relationships that could be construed as a potential conflict of interest.
