# Supplementary material for: LAM Genes Contribute to Environmental Stress Tolerance but Sensibilize Yeast Cells to Azoles
Source: Front Microbiol. 2020 Jan 28;11:38. doi: 10.3389/fmicb.2020.00038 (PMC6997477; doi:10.3389/fmicb.2020.00038)
Supplement: Supplementary file 1 [file Data_Sheet_1.docx]

**Supplementary information**

*LAM* genes contribute to environmental stress tolerance but sensibilise yeast cells to azoles

## S.S.Sokolov^1^, M.A.Vorobeva^2^, A.I.Smirnova^1^, E.A.Smirnova^1^, N.I.Trushina^1,3^, K.V.Galkina^1,2^, F.F.Severin^1^, D.A.Knorre^1,4^

^1^Belozersky Institute of Physico-Chemical Biology, Moscow State University, Leninskiye Gory 1–40, Moscow, 119991, Russia.

^2^Faculty of Bioengineering and Bioinformatics, Lomonosov Moscow State University, Leninskiye Gory 1-73, Moscow 119991, Russia

^3^Department of Neurobiology, University of Osnabrück, Barbarastraße 11, D-49076 Osnabrück, Germany

^4^Institute of Molecular Medicine, Sechenov First Moscow State Medical University, Moscow, 119991, Russia

**
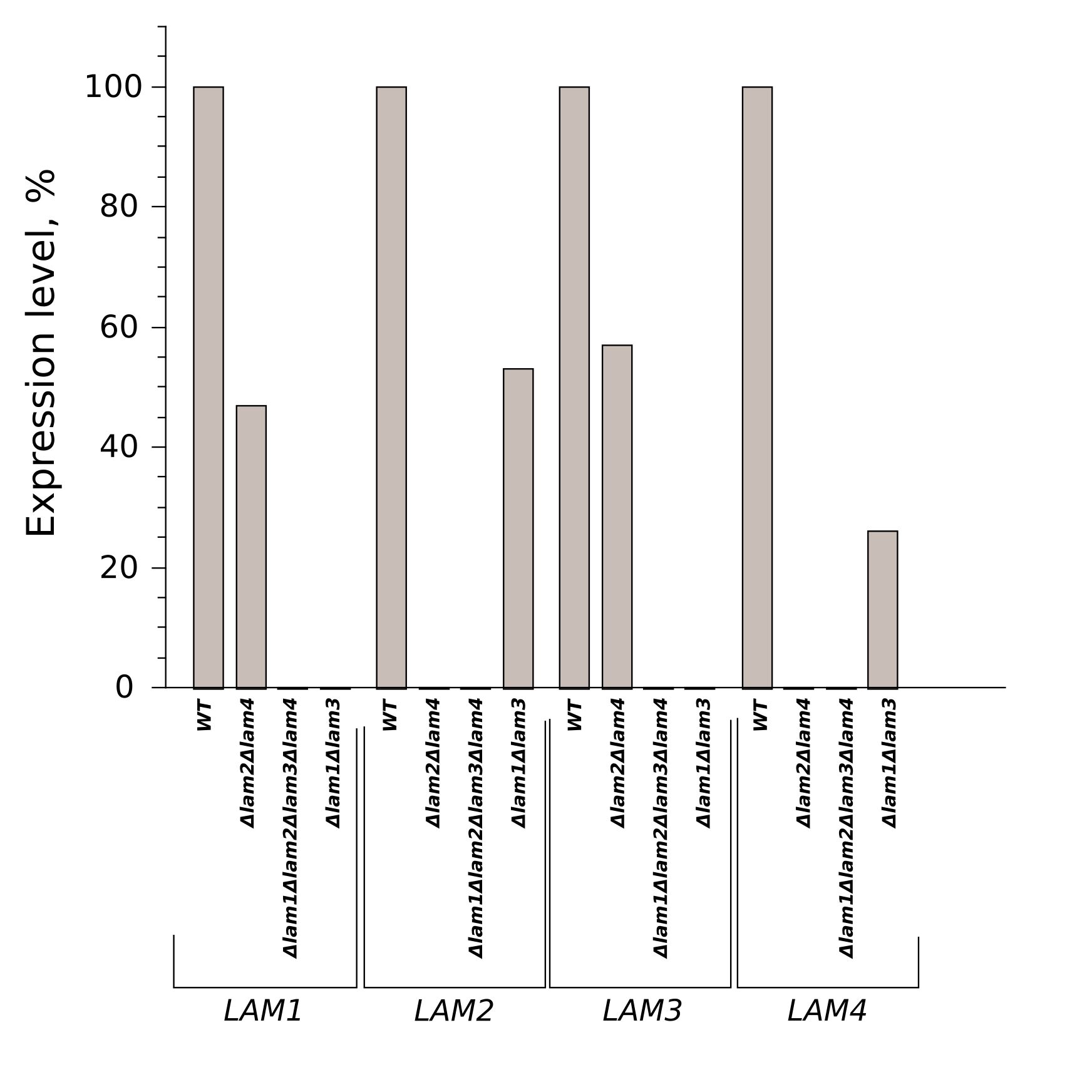
**

**Figure S1.**  mRNA levels of *LAM1-LAM4* genes in the double and quadruple deletion strains. All mRNA levels were normalized to *ACT1* levels, the value in the untreated wild-type cells (WT) was set to 100%. (n = 1)

**Supplementary text 1. Quantitative reverse transcription PCR (RT-qPCR) analysis.**

RNA was isolated from yeast cells using the hot formamide extraction method described in (Shedlovskiy et al. 2017). RNA quality and quantity were assessed by spectrophotometry. cDNA was synthesized by annealing 2 μg of RNA with 0.1 μg of random hexamers and 0.1 μg of Oligo-dT using Superscript III reverse-transcriptase (Thermo Fisher Scientific) for 1 hour at 42 °C. RT-qPCR was carried out using the CFX96 Touch™ Real-Time PCR Detection System (Bio-Rad, Hercules, CA, USA). We used designed primer sequences for LAM genes (Table S1). For the detection of the target genes, Eva Green master mix (Syntol, Russia) was used according to the manufacturer’s instructions. The thermal profile for EVA Green RT-qPCR included an initial heat-denaturing step at 95 °C for 3 minutes, 40 cycles at 95 °C for 15 seconds, an annealing step for 30 seconds and 72 °C for 30 seconds, coupled with fluorescence measurements. Following amplification, the melting curves of the PCR products were monitored to determine the specificity of the amplification. The PCR efficiency (E) was calculated according to the equation E = 10(−1/slope) based on the standard curves. Target mRNA levels were normalized to the reference gene ACT1.

Shedlovskiy D, Shcherbik N, Pestov DG. One-step hot formamide extraction of RNA from Saccharomyces cerevisiae. RNA Biol. 2017;14: 1722–1726.


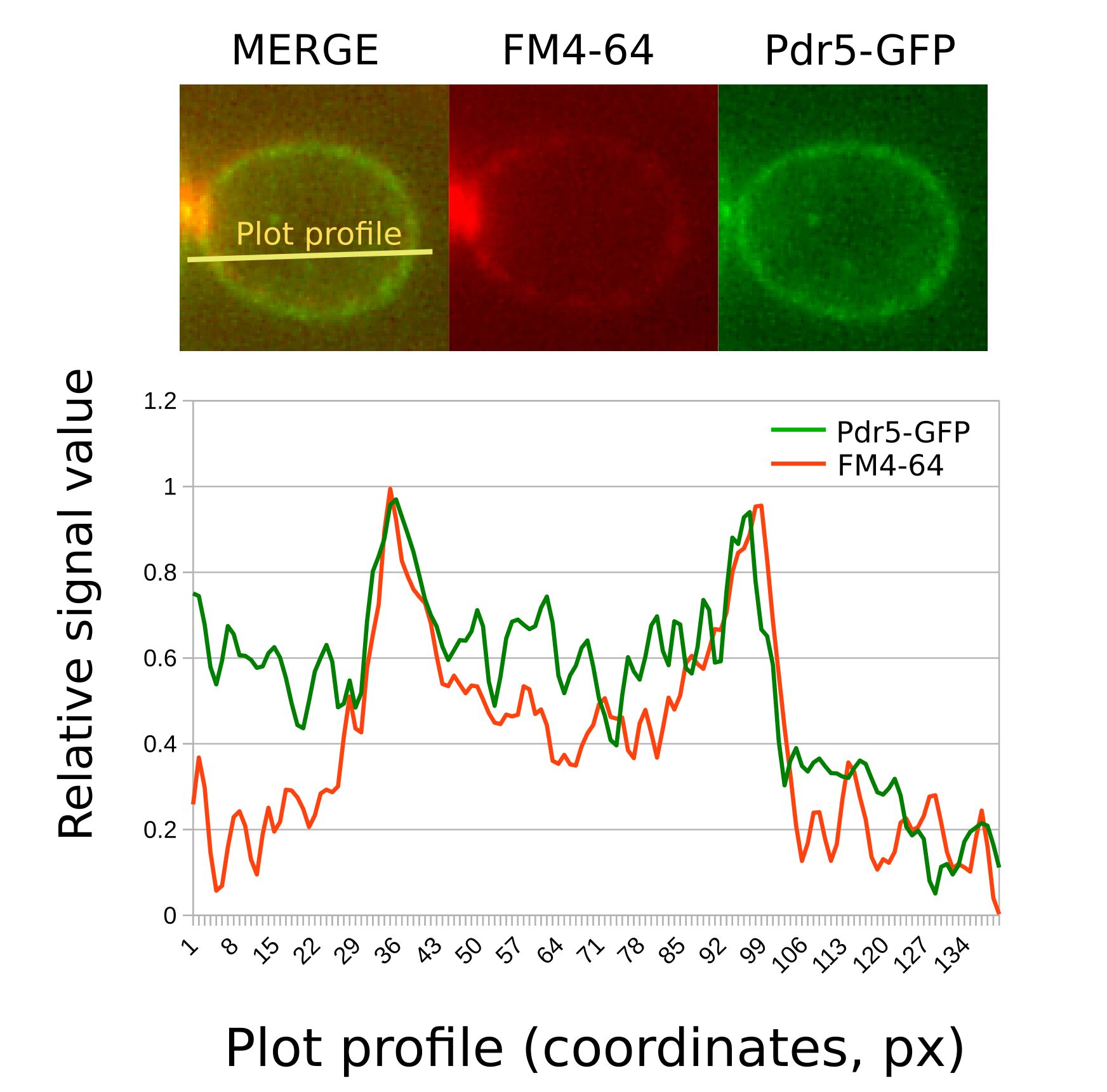


Figure S2. Colocalization of FM4-64 (0.8 µM) with Pdr5-GFP in Δlam1Δlam2Δlam3Δlam4 cells preincubated for one hour with pentachlorophenol, 20 µM and deoxyglucose, 1%. Cells were analyzed instantly after the addition of FM4-64. Upper panel, a representative photograph. Lower panel, plot intensity profiles of FM4-64 and Pdr5-GFP signals across an individual yeast cell.

Table S1.

Oligonucleotide primers used in this study

| **Primer** | **Sequence** |
| --- | --- |
| LAM1 Sense | CACAAGCCAGCCACAGTATC |
| LAM1 AntiSense | GCACCACACCGATGAATATCTT |
| LAM2 Sense | ACAATGACGACGATGGAGAC |
| LAM2 AntiSense | AATCAGCAGTGGTTGGAGAG |
| LAM3 Sense | ACCGATTGGAGTGAATATAC |
| LAM3 AntiSense | ACTAATGACTTGAACTGTAGAT |
| LAM4 Sense | ATGACGATTACGATGATGATTACG |
| LAM4 AntiSense | TTCCTATGCTGACGCTTGA |
| ACT1 Sense | GAATTGAGAGTTGCCCCAGA |
| ACT1 AntiSense | GGCTTGGATGGAAACGTAGA |
